# Knockdown of the HDAC1 Promotes the Directed Differentiation of Bone Mesenchymal Stem Cells into Cardiomyocytes

**DOI:** 10.1371/journal.pone.0092179

**Published:** 2014-03-31

**Authors:** Dong-feng Lu, Ying Wang, Zi-zhuo Su, Zhao-hua Zeng, Xiao-wen Xing, Zhi-yu He, Chunxiang Zhang

**Affiliations:** 1 Department of Cardiology, the First Affiliated Hospital of Guangzhou Medical University, Guangzhou, Guangdong, China; 2 Department of Pharmacology and Cardiovascular Research Center, Rush Medical college of Rush University, Chicago, Illinois, United States of America; Northwestern University, United States of America

## Abstract

Failure of the directed differentiation of the transplanted stem cells into cardiomyocytes is still a major challenge of cardiac regeneration therapy. Our recent study has demonstrated that the expression of histone deacetylase 1 (HDAC1) is decreased in bone mesenchymal stem cells (BMSCs) during their differentiation into cardiomyocytes. However, the potential roles of HDAC1 in cardiac cell differentiation of BMSCs, as well as the mechanisms involved are still unclear. In current study, the expression of HDAC1 in cultured rat BMSCs is knocked down by lentiviral vectors expressing HDAC1-RNAi. The directed differentiation of BMSCs into cardiomyocytes is evaluated by the expression levels of cardiomyocyte-related genes such as GATA-binding protein 4 (GATA-4), Nirenberg, Kim gene 2 homeobox 5 (Nkx2.5), cardiac troponin T (CTnT), myosin heavy chain (MHC), and connexin-43. Compared with that in control BMSCs, the expression of these cardiomyocyte-related genes is significantly increased in these HDAC1 deficient stem cells. The results suggest that HDAC1 is involved in the cardiomyocyte differentiation of BMSCs. Knockdown of the HDAC1 may promote the directed differentiation of BMSCs into cardiomyocytes.

## Introduction

Bone mesenchymal stem cells (BMSCs) are non-hematopoietic stem cells in bone marrow. BMSCs are ideal cell-based regeneration therapy in the treatment of cardiovascular disease because these cells exhibit various advantageous traits, including ready accessibility, ease of amplification, low immunogenicity, and amenability to the introduction of exogenous genes and other genetic modifications [1], [2].

Despite the current optional approaches, failure of the directed differentiation of the transplanted stem cells into cardiomyocytes is still a major challenge of cardiac regeneration therapy [3]. This has highlighted the importance and urgency of studying the novel mechanisms of this critical cellular process and exploring new therapeutic options to improve our cardiac regeneration therapy.

Recently, an increasing number of studies have revealed that the epigenetic modification of histone acetylation may play an important role in the transdifferentiation processes of adult stem cells including their directed cardiac cell differentiation [4], [5], [6], [7]. In a pilot study, we have identified that the expression of histone deacetylase 1 (HDAC1) in BMSCs is significantly decreased during their differentiation into cardiomyocytes. However, until now, the potential role of HDAC1 in the directed differentiation of the transplanted stem cells into cardiomyocytes is still unclear. In current study, the expression of HDAC1 in cultured rat BMSCs is knocked down by lentiviral vectors expressing HDAC1-RNAi and the directed differentiation of BMSCs into cardiomyocytes is evaluated.

## Materials and Methods

### 1.1. Experimental animals

One-month-old male Sprague-Dawley (SD) rats, weighing 80 to 110 g, were purchased from the Guangdong Medical Laboratory Animal Center. All protocols were approved by the Institutional Animal Care and Use Committee at the Guangzhou Medical University and were consistent with the Guide for the Care and Use of Laboratory Animals (NIH publication 85-23, revised 1985).

### 1.2. The isolation, culture and identification of BMSCs

SD rat BMSCs were isolated and cultured using the whole bone marrow adherence method described previously [8]. Briefly, SD rats were sacrificed, and their femora and tibiae were rapidly stripped. A 5 ml syringe loaded with an appropriate quantity of complete BMSC culture medium (Dulbecco's modified Eagle's medium/Ham's F12 nutrient mixture (DMEM/F12) with 10% FBS, 100 U/ml penicillin, and 100 U/ml streptomycin) was inserted into the metaphysis of each bone, and was utilised to flush bone marrow cells with culture medium. The isolated BMSCs were centrifuged at 1000 rpm for 10 minutes, resuspended in complete medium, and cultured. The culture medium was changed every 2–3 days. After reaching 80% confluence, cultured adherent cells were trypsinised with a 0.25% trypsin solution and passaged. The percentages of cells that were positive for the BMSC surface markers of CD34, CD45, CD29, CD44, and CD90 (Santa Cruz) were analysed by flow cytometry. For all experiments, rat BMSCs from passages 3 to 5 (P3 to P5 cells) were used.

### 1.3. The construction and screening of the optimal HDAC1-RNAi lentiviral vector

Based on the sequence information of the HDAC1 gene, four short hairpin RNA (shRNA) sequences and one negative control (NC) sequence were designed. The shRNA expression sequences targeting HDAC1 were ligated into the lentiviral vector pGCSIL-GFP to obtain recombinant pGCSIL-GFP-shHDAC1 plasmids. After the plasmid sequences had been verified, the recombinant plasmids and the lentiviral packaging plasmids pHelper 1.0 and pHelper 2.0 were cotransfected into 293T cells using Lipofectamine 2000. Virus was collected from the culture supernatant, and the viral titre of this solution was determined by serial dilution.

The packaged pGCSIL-GFP-NC lentivirus was used to infect BMSCs at MOIs of 0, 1, 10, and 100. Fluorescence microscopy was utilised to detect the expression of fluorescent proteins after 12 h, 24 h, 48 h, and 72 h of infection. Flow cytometry (at a wavelength of 488 nm) was used to determine infection efficiency and thereby screen for the MOI value and infection time that produced maximal infection efficiency.

Five groups of packaged viruses were used to infect BMSCs, and the BMSCs were collected after 48 hours of infection. Uninfected BMSCs were utilised as a normal control group. To screen for the virus with the best efficiency at silencing HDAC1, real-time quantitative reverse transcription polymerase chain reaction (RT-qPCR) was used to determine the HDAC1 mRNA expression levels for each group, and Western blotting was used to determine the HDAC1 protein expression levels for each group.

### 1.4. RT-PCR

TRIzol was used to extract total RNA samples from cells. Subsequently, a TaKaRa transcription kit was utilised for the reverse transcription (RT) of these samples; in this reaction, the samples were incubated in a PCR thermocycler at 37°C for 15 minutes and then at 85°C for 5 seconds. The RT-PCR amplifications were performed with the TaKaRa PrimeScript II 1st Strand cDNA Synthesis Kit (D6210A), using glyceraldehyde 3-phosphate dehydrogenase (GAPDH) as an internal reference. The amplification included the following reaction stages: stage I (initial denaturation), which involved an incubation at 95°C for 30 sec; stage II (30 cycles of PCR amplification), which involved 30 cycles of incubation at 95°C for 5 sec, 60°C for 3 sec, and 72°C for 30 s; and stage III (melting curve analysis), which involved an incubation at 72°C for 10 min followed by an incubation at 16°C for 10 min. The primers for various genes were designed using the Primer 5.0 software package and synthesised by TaKaRa Biotechnology. In particular, the following specific primers were synthesised: HDAC1, 149 bp, F: 5′-TCA CCG AAT CCG AAT GAC TCA TAA-3′, R: 5′- CTG GGC GAA TAG AAC GCA AGA-3′; GAPDH, 143 bp, F: 5′- GGC ACA GTC AAG GCT GAG AAT G-3′, R: 5′- ATG GTG GTG AAG ACG CCA GTA-3′;GATA binding protein 4 (GATA-4), 180 bp, F: 5′- AAC GGA AGC CCA AGA ATC TGA-3′, R: 5′- AGC TGC TGT GCC CAT AGT GAG-3′; Nirenberg and Kim gene 2 homeobox 5 (Nkx2.5), 178 bp, F: 5′- GTC TCA ACG CCT ACG GCT AC-3′, R: 5′- CTC TGC ACC GTG TTC AAG TC-3′;cardiac troponin T(CTnT), 162 bp, F: 5′- AGA TTG CGA AGC AGG AGA TG -3′,R: 5′- CAT CCA CTT TGT CCA CAC GA -3′;myosin heavy chain (MHC), 151 bp, F: 5′- GAG CAG GAG CTG ATC GAG AC -3′, R: 5′- CCT CTG CGT TCC TAC ACT CC -3′;and connexin-43, 165 bp, F: 5′- TCG TGT CTG TGC CCA CCC TC -3′, R:5′- TCC CGT ACT TGA ACT TCT TGA TTT -3′. Based on the amplification results, the 2-ΔΔCT method was used to calculate the relative multiple of the starting copy number that existed in the template from each experimental group and thereby indicate the relative differences in mRNA levels among the experimental groups.

### 1.5. Western blotting

At least 1×10^7^ cells were collected for the extraction of nucleoproteins, which was performed as described in the DBI nucleoprotein extraction kit. Protein concentrations were determined with the bicinchoninic acid (BCA) assay. Samples of 16 μg of nucleoproteins were separated on a 10% sodium dodecyl sulphate-polyacrylamide gel electrophoresis (SDS-PAGE) gel and subsequently electrotransferred onto polyvinylidene difluoride (PVDF) membranes for 90 min at 4°C and 290 mA. After these membranes were blocked in 5% non-fat dry milk for 90 min, they were incubated with a diluted anti-HDAC1 primary antibody (1∶500 dilution, Abcam) (in a solution that contained 2% non-fat dry milk) and incubated at 4°C overnight. Subsequently, the membranes were washed with phosphate-buffered saline (PBS) or Tris-buffered saline with Tween 20 (TBST), using sufficient saline solution to cover the PVDF membrane for each wash. The first wash step was performed over the course of 15 min, whereas each of the next three washes were 5 min in duration. Following these washes, the membranes were incubated with 5 ml of 1∶5000 diluted goat anti-rabbit secondary antibody (in a solution that contained 2% non-fat dry milk) for 1 h at room temperature. An enhanced chemiluminescence (ECL) solution was prepared; the membranes were incubated in this ECL solution in a darkroom for 5 min, and images of the membranes were then digitally developed. The relative expression level of a target protein was quantified by dividing the greyscale value of the target protein band by the greyscale value of the band corresponding to GAPDH, the protein that was utilised as an internal reference.

### 1.6. Statistical analysis

Statistical analyses were performed using the SPSS 13.0 statistical software package, and the data were expressed as means ± standard deviation. Comparisons between two groups were performed using the independent samples t-test, and comparisons among multiple groups were performed using the one-way analyses of variance; P<0.05 was regarded as statistically significant.

## Results

### 1. The morphological characteristics and identification of BMSCs in vitro

**Figure 1:**
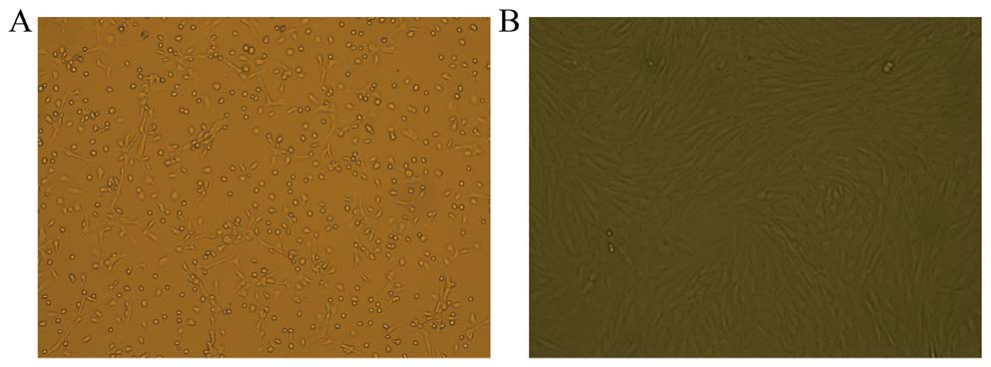
Morphological observations of BMSCs. A. Primary cultured BMSCs (100× magnification). B. BMSCs at passage 3 (P3) (100× magnification).

**Figure 2:**
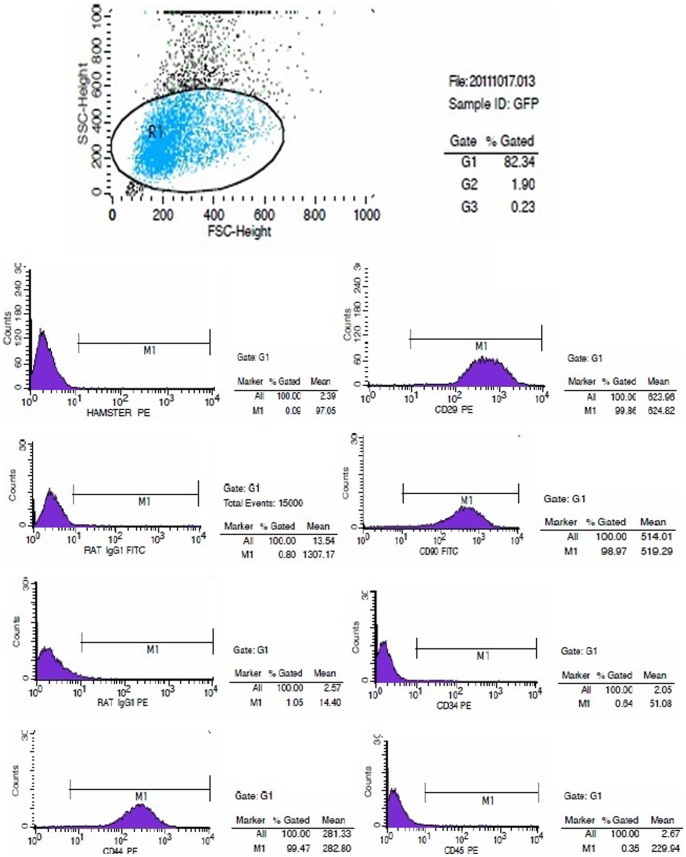
Flow cytometry observations of BMSC surface markers. The expressed levels of the hematopoietic markers CD34 and CD45, and the mesenchymal cell surface markers, CD29, CD44, and CD90 in BMSCs.

At the early stage of primary BMSC cultures, the cells were round. After 24 h of culture, the BMSCs had become adherent and had elongated to adopt fusiform or triangular shapes. After 3–4 d, cells had entered the logarithmic growth phase and exhibited clonal proliferation. These BMSC colonies fused after 9–10 d of culture; at this time, most of the cells exhibited the morphology of flattened long spindles, whereas a small proportion of cells were either triangle- or polygon-shaped. Cells became adherent relatively rapidly after they were passaged. Passaged cells demonstrated a fibroblast-like morphology and an increased volume relative to primary cells. The passaged cells underwent rapid clonal proliferation, requiring only 3–4 d to reach 80% confluence; at this stage, the cells were either re-passaged or cryopreserved. As the number of passages of cells increased, cells became increasingly purified, and the observed cell morphologies became more uniform. Moreover, the BMSCs exhibited a polar arrangement that was swirling in nature or reminiscent of schools of fish (Figure 1). BMSCs at three passages were examined by flow cytometry. The results of these analyses indicated that these BMSCs expressed low levels of the hematopoietic markers CD34 (0.64%) and CD45 (0.35%) but high levels of various stromal and mesenchymal cell surface markers, including CD29 (99.86%), CD44 (99.47%), and CD90 (98.97%) (Figure 2).

### 2. The construction and screening of the optimal HDAC1-RNAi lentiviral vector

#### 2.1. Sequencing verification of recombinant plasmids

**Figure 3:**
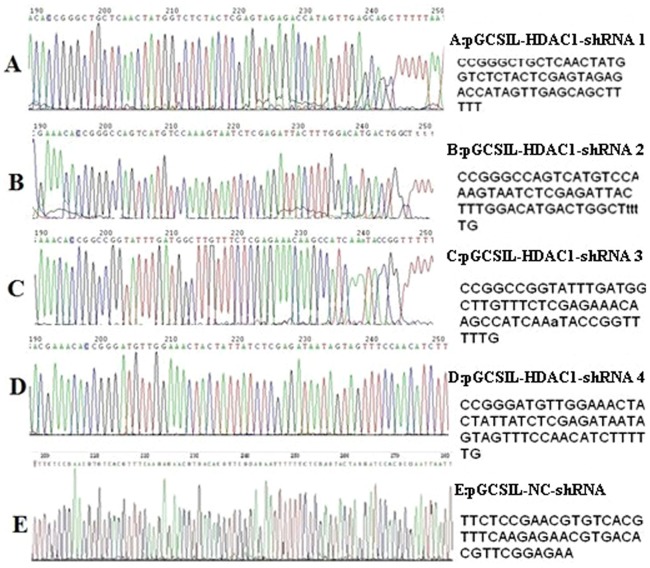
Colour sequencing maps of the recombinant plasmids. The positive clones were designated pGCSIL-HDAC1-shRNA1 through pGCSIL-HDAC1-shRNA4, and the NC vector was known as pGCSIL-NC-shRNA.

The recombinant vectors of this experiment were sequenced by Shanghai GeneChem. The positive clones were designated pGCSIL-HDAC1-shRNA1 through pGCSIL-HDAC1-shRNA4, and the NC vector was known as pGCSIL-NC-shRNA. The sequencing results indicated that the shRNA expression template was successfully constructed based on the pGCSIL-GFP vector and that the sequences of interest in each vector were completely correct and identical to the designed target sequence (Figure 3).

#### 2.2. The infection of target cells: preliminary experiments and screening for optimal MOI conditions

**Figure 4:**
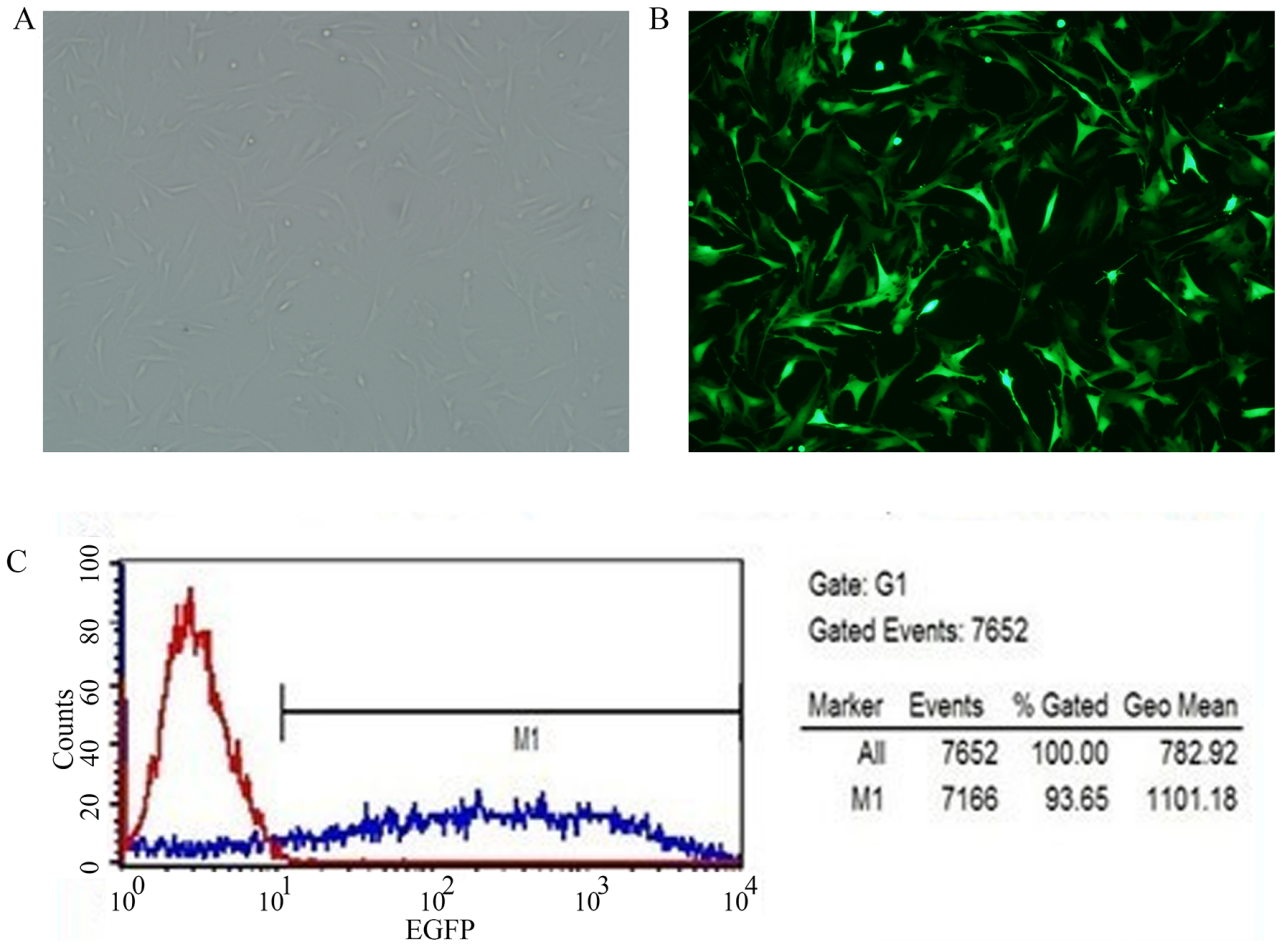
Observations of the efficiency of the lentiviral infection of BMSCs. A, Inverted microscopy of BMSCs infected with lentivirus for 48(100× magnification). B, Fluorescence microscopy of BMSCs infected with lentivirus for 48 h (100× magnification). C, Flow cytometric measurements of BMSC infection efficiency (93.65%).

Expression of enhanced green fluorescent protein (EGFP) could be observed at 12 h after BMSCs had been infected with the pGCSIL-GFP-NC lentivirus; the EGFP expression levels peaked at 48 h after infection, although strong green fluorescence was sustained through 72 h after infection. In particular, compared with the other examined groups, the ENi.S+5 μg/ml polybrene group exhibited a significantly higher number of fluorescent cells. Under optimal infection conditions, at an MOI of between 1 and 10, an infection efficiency of less than 70% was observed. By contrast, at an MOI of 100, an infection efficiency of 90% or more was observed; moreover, cells exhibited strong growth and no significant differences in cell morphology relative to the normal control group. Flow cytometry revealed an EGFP-positive rate of 93.65% among these infected cells (Figure 4).

#### 2.3. HDAC1 mRNA expression determined by RT-PCR

**Figure 5:**
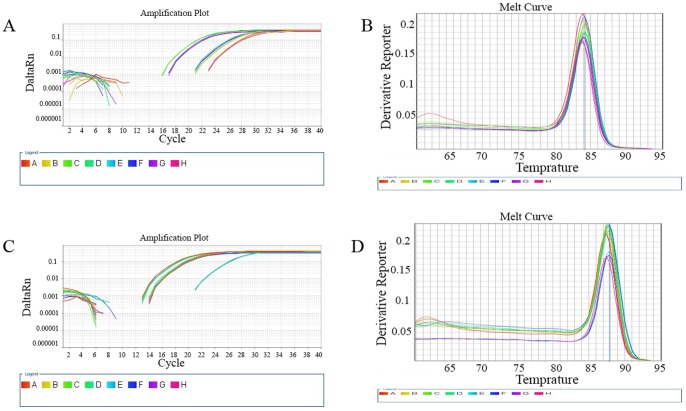
Amplification and melting curves for HDAC1 and GAPDH. A, HDAC1 amplification curve. B, HDAC1 melting curve. C, GAPDH amplification curve. D, GAPDH melting curve.

**Figure 6:**
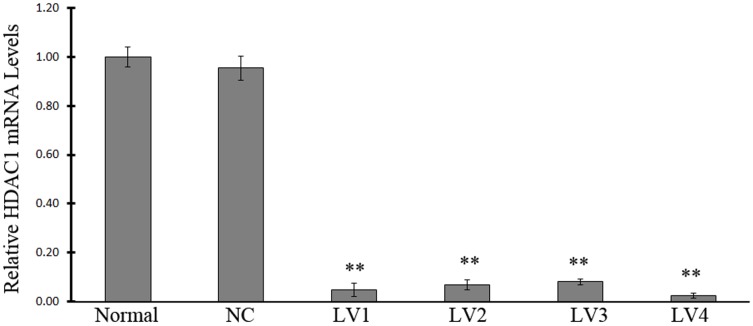
The relative HDAC1 mRNA levels. The cells were infected with vehicle (normal control), NC vector (NC, pGCSIL-NC-shRNA), LV-HDAC1-shRNA1 (LV1), LV-HDAC1-shRNA2 (LV2), LV-HDAC1-shRNA3 (LV3), or LV-HDAC1-shRNA4 (LV4). Note: n = 6; ** P<0.01, compared with that in NC group.

RT-PCR results revealed that although HDAC1 mRNA could be detected in samples from all groups, significantly lower HDAC1 mRNA expression levels were detected in the groups infected with an interference virus compared with those in either the normal control group or the NC group (P<0.01, Figure 5, Figure 6). In particular, the normal control group exhibited an HDAC1 mRNA expression level that was 32, 11, 13, and 96 times higher than the HDAC1 mRNA expression levels of the LV-HDAC1-shRNA1, LV-HDAC1-shRNA2, LV-HDAC1-shRNA3, and LV-HDAC1-shRNA4 interference groups, respectively. No significant differences in HDAC1 mRNA expression were found between the normal control group and the NC group. Although all the four vectors that were able to successfully inhibit the HDAC expression, the inhibitory effect of LV-HDAC1-shRNA1 and LV-HDAC1-shRNA4 was more efficient compared with that of LV-HDAC-shRNA2 and LV-HDAC1-shRNA3.

#### 2.4. HDAC1 expression determined by Western blotting

**Figure 7:**
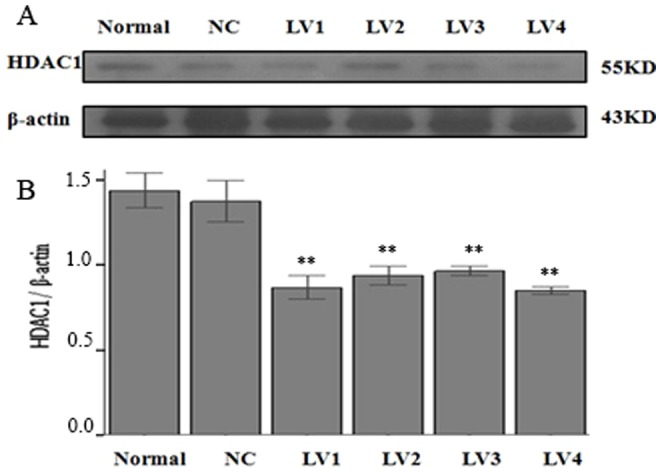
The HDAC1 protein levels. The cells were infected with vehicle (normal control), NC vector (NC, pGCSIL-NC-shRNA), LV-HDAC1-shRNA1 (LV1), LV-HDAC1-shRNA2 (LV2), LV-HDAC1-shRNA3 (LV3), or LV-HDAC1-shRNA4 (LV4). Note: n = 6; ** P<0.01, compared with that in NC group.

The results of western blotting indicated that although HDAC1 protein was expressed in samples from all groups, significantly lower HDAC1 protein expression levels were detected in all the groups infected with an interference virus compared with those of the normal control group or the NC group (P<0.01, Figure 7). In particular, the normal control group exhibited an HDAC1 protein expression level that was 1.66 times, 1.53 times, 1.50 times, and 1.70 times higher than those in LV-HDAC1-shRNA1, LV-HDAC1-shRNA2, LV-HDAC1-shRNA3, and LV-HDAC1-shRNA4 interference groups, respectively. Thus, the inhibitory efficiencies of these interference groups at the protein level were 40%, 35%, 32%, and 41%, respectively. Based on the results, LV-HDAC1-shRNA4 vector was selected to be used in our subsequent experiments.

### 3. RT-PCR detection of genes related to myocardial development and structure

The expression levels of genes related to myocardial development and structure were detected in BMSCs infected with LV-HDAC1-shRNA4. The genes detected in this study included Nkx2.5, GATA-4, MHC, connexin-43, and CTnT. As shown in Table 1, the expression of these 5 genes was significantly higher in BMSCs infected with the lentivirus compared with that in cells from either the normal control group or the NC group (P<0.05). No significant difference was found between the normal control group and the NC group with respect to the expression of the 5 detected genes.

**Table 1:** Myocardial markers expression levels in experimental groups (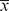
±sd).

Markers	Normal	LV-NC-shRNA	LV-HDAC4-shRNA4
Nkx2.5	1.000±0.023	0.9107±0.094	4.898±1.754*
GATA-4	1.000±0.026	0.9913±0.039	76.455±26.624*
MHC	1.000±0.021	0.900±0.059	52.101±9.366*
Connexin-43	1.000±0.025	1.102±0.032	37.131±3.589*
CTnT	1.000±0.016	1.076±0.076	41.183±13.193*

Note: n = 6; * P<0.05, compared with that in LV-NC-shRNA group.

## Discussion

In our recent pilot study, we have found that the expression level of HDAC1 is significantly decreased in BMSCs after co-cultured with cardiomyocytes (Data not shown). Moreover, nascent cardiomyocytes exhibite low levels of HDAC1 expression. These initial results suggest that HDAC1 gene expression might be related to the directed differentiation of BMSCs into cardiomyocytes. We thus hypothesize that the HDAC1 might be a negatively regulator in cardiac cell differentiation from BMSCs. To test our hypothesis, we constructed a shRNA eukaryotic expression vector that specifically silenced the HDAC1 gene by RNAi. We found that HDAC1-shRNA could significantly reduce HDAC1 expression in BMSCs; this reduction in HDAC1 expression in BMSCs significantly increased the expression of cardiac-specific genes, such as Nkx2.5, GATA-4, MHC, connexin-43, and CTnT. Thus, the specific inhibition of HDAC1 gene in BMSCs is able to increase the differentiation of BMSCs into cardiac cell phenotype.

Directed differentiation of stem cells is a complex process in which multiple genes and signalling pathways are involved [9]. Recently, epigenetic modification of histone acetylation has been identified to play impartment roles in this stem cell process [4], [5], [6], [7]. Indeed, rat BMSCs treated with the HDAC inhibitors such as suberoylanilide hydroxamic acid (SAHA) and trichostatin A (TSA) exhibit a significant increase in the expression of cardiomyocyte-specific genes, including GATA4, Nkx2.5, and Mef2c [5], [10]. However, both SAHA and TSA are non-specific inhibitors of HDACs that could suppress multiple HDAC subtypes in addition to HDAC1. In addition, SAHA and TSA treatments promote not only the differentiation of BMSCs into cardiomyocyte-like cells but also the differentiation of BMSCs into endothelial cells [11], hepatocytes [12], chondrocytes [13], and adipocytes [14], among other cell types. However, the detailed information regarding the roles of HDAC subtypes in the differentiation of BMSCs into myocardial phenotypes has not yet been reported. In this study, we identified, via the shRNA approach, that HDAC1 is a critical regulator for the directed differentiation of BMSCs into myocardial phenotypes. The results of our study are consistent with the studies in other stem cell lines. For example, Liu et al. [15] and Dovey et al. [16] have reported that the in vitro induction of specific deficiencies in HDAC1 expression in P19CL6 cells and embryonic stem cells could promote the differentiation of stem cells into myocardial phenotypes.

The molecular mechanisms involved in HDAC1-mediated effect on the directed differentiation of BMSCs into myocardial phenotypes are currently unclear. In embryonic stem cells, Hoxha et al. [17] reported that HDAC1 regulates the phenotypic differentiation of embryonic stem cells into cardiomyocytes by controlling the expression of sex-determining region Y-box 17 (SOX17) and bone morphogenetic protein 2 (BMP2). However, because of ethical concerns and other issues, embryonic stem cells and induced pluripotent stem cells might not be used for clinical stem cell transplantation in the near future. The elucidation of the downstream pathways involved in the HDAC-induced the phenotypic differentiation of BMSCs into myocardial phenotypes should be identified in future study. Moreover, the roles of other HDAC subtypes in cardiac cell differentiation from BMSCs merit additional investigation in the future.

In summary, we have identified that the specific inhibition of HDAC1 gene expression in BMSCs by RNAi causes an increase in the mRNA expression levels of genes related to myocardial development and structure. HDAC1 may play an important role in the directed differentiation of BMSCs into cardiomyocytes.
